# Supplementary material for: Diagnosis of Acute Aortic Syndromes on Non-Contrast CT Images with Radiomics-Based Machine Learning
Source: Biology (Basel). 2023 Feb 21;12(3):337. doi: 10.3390/biology12030337 (PMC10045362; doi:10.3390/biology12030337)
Supplement: Supplementary file 1 [file biology-12-00337-s001.zip › SupplementaryS1.pdf]

fature	ICC
original_shape_Elongation	0.96933716
original_shape_Flatness	0.97321227
original_shape_LeastAxisLength	0.95453729
original_shape_MajorAxisLength	0.96571729
original_shape_Maximum2DDiameterColumn	0.94864554
original_shape_Maximum2DDiameterRow	0.87055306
original_shape_Maximum2DDiameterSlice	0.96955988
original_shape_Maximum3DDiameter	0.94059512
original_shape_MeshVolume	0.97420628
original_shape_MinorAxisLength	0.97386506
original_shape_Sphericity	0.60373626
original_shape_SurfaceArea	0.93269739
original_shape_SurfaceVolumeRatio	0.88980301
original_shape_VoxelVolume	0.97417295
original_firstorder_10Percentile	0.75971667
original_firstorder_90Percentile	0.8402117
original_firstorder_Energy	0.97574585
original_firstorder_Entropy	0.83627987
original_firstorder_InterquartileRange	0.89351082
original_firstorder_Kurtosis	0.49456345
original_firstorder_Maximum	0.78927625
original_firstorder_MeanAbsoluteDeviation	0.60762744
original_firstorder_Mean	0.74496639
original_firstorder_Median	0.99010554
original_firstorder_Minimum	0.75604043
original_firstorder_Range	0.83360133
original_firstorder_RobustMeanAbsoluteDeviation	0.8993737
original_firstorder_RootMeanSquared	0.69265353
original_firstorder_Skewness	0.70689266
original_firstorder_TotalEnergy	0.97574585
original_firstorder_Uniformity	0.91389115
original_firstorder_Variance	0.48904949
original_glcm_Autocorrelation	0.83974471
original_glcm_JointAverage	0.75678477
original_glcm_ClusterProminence	0.25148765
original_glcm_ClusterShade	0.22963459
original_glcm_ClusterTendency	0.42285872
original_glcm_Contrast	0.52047802
original_glcm_Correlation	0.78049838
original_glcm_DifferenceAverage	0.79085413
original_glcm_DifferenceEntropy	0.86626159
original_glcm_DifferenceVariance	0.49622624
original_glcm_JointEnergy	0.96081574
original_glcm_JointEntropy	0.86871547
original_glcm_Imc1	0.80489478
original_glcm_Imc2	0.78479031
original_glcm_Idm	0.94389692
original_glcm_Idmn	0.94088622

original_glcm_Id	0.9484771
original_glcm_Idn	0.94233499
original_glcm_InverseVariance	0.99065029
original_glcm_MaximumProbability	0.98597215
original_glcm_SumEntropy	0.83455601
original_glcm_SumSquares	0.43979375
original_glrlm_GrayLevelNonUniformity	0.97593505
original_glrlm_GrayLevelNonUniformityNormalized	0.81290751
original_glrlm_GrayLevelVariance	0.43482506
original_glrlm_HighGrayLevelRunEmphasis	0.84158129
original_glrlm_LongRunEmphasis	0.4205608
original_glrlm_LongRunHighGrayLevelEmphasis	0.96423188
original_glrlm_LongRunLowGrayLevelEmphasis	0.75789269
original_glrlm_LowGrayLevelRunEmphasis	0.85914822
original_glrlm_RunEntropy	0.81888613
original_glrlm_RunLengthNonUniformity	0.95018258
original_glrlm_RunLengthNonUniformityNormalized	0.94090402
original_glrlm_RunPercentage	0.97603079
original_glrlm_RunVariance	0.9803175
original_glrlm_ShortRunEmphasis	0.93101033
original_glrlm_ShortRunHighGrayLevelEmphasis	0.86757435
original_glrlm_ShortRunLowGrayLevelEmphasis	0.8665288
original_glszm_GrayLevelNonUniformity	0.97101726
original_glszm_GrayLevelNonUniformityNormalized	0.8483193
original_glszm_GrayLevelVariance	0.67336399
original_glszm_HighGrayLevelZoneEmphasis	0.84805795
original_glszm_LargeAreaEmphasis	0.76903721
original_glszm_LargeAreaHighGrayLevelEmphasis	0.63266807
original_glszm_LargeAreaLowGrayLevelEmphasis	0.49546754
original_glszm_LowGrayLevelZoneEmphasis	0.81411166
original_glszm_SizeZoneNonUniformity	0.8702108
original_glszm_SizeZoneNonUniformityNormalized	0.94195528
original_glszm_SmallAreaEmphasis	0.93837527
original_glszm_SmallAreaHighGrayLevelEmphasis	0.8822193
original_glszm_SmallAreaLowGrayLevelEmphasis	0.71784079
original_glszm_ZoneEntropy	0.80492024
original_glszm_ZonePercentage	0.89926955
original_glszm_ZoneVariance	0.76898753
original_gldm_DependenceEntropy	0.85995251
original_gldm_DependenceNonUniformity	0.97822336
original_gldm_DependenceNonUniformityNormalized	0.94528816
original_gldm_DependenceVariance	0.97076181
original_gldm_GrayLevelNonUniformity	0.98046551
original_gldm_GrayLevelVariance	0.48911563
original_gldm_HighGrayLevelEmphasis	0.84040732
original_gldm_LargeDependenceEmphasis	0.98519768
original_gldm_LargeDependenceHighGrayLevelEmphasis	0.66921583
original_gldm_LargeDependenceLowGrayLevelEmphasis	0.80619084
original_gldm_LowGrayLevelEmphasis	0.8602618

original_gldm_SmallDependenceEmphasis	0.90709933
original_gldm_SmallDependenceHighGrayLevelEmphasis	0.86687693
original_gldm_SmallDependenceLowGrayLevelEmphasis	0.84450344
log-sigma-1-0-mm-3D_firstorder_10Percentile	0.88901004
log-sigma-1-0-mm-3D_firstorder_90Percentile	0.98353459
log-sigma-1-0-mm-3D_firstorder_Energy	0.97509678
log-sigma-1-0-mm-3D_firstorder_Entropy	0.93686604
log-sigma-1-0-mm-3D_firstorder_InterquartileRange	0.96486417
log-sigma-1-0-mm-3D_firstorder_Kurtosis	0.4653475
log-sigma-1-0-mm-3D_firstorder_Maximum	0.97769097
log-sigma-1-0-mm-3D_firstorder_MeanAbsoluteDeviation	0.87880924
log-sigma-1-0-mm-3D_firstorder_Mean	0.64898322
log-sigma-1-0-mm-3D_firstorder_Median	0.95767086
log-sigma-1-0-mm-3D_firstorder_Minimum	0.70632426
log-sigma-1-0-mm-3D_firstorder_Range	0.83303237
log-sigma-1-0-mm-3D_firstorder_RobustMeanAbsoluteDeviation	0.95419371
log-sigma-1-0-mm-3D_firstorder_RootMeanSquared	0.70213781
log-sigma-1-0-mm-3D_firstorder_Skewness	0.4537035
log-sigma-1-0-mm-3D_firstorder_TotalEnergy	0.97509678
log-sigma-1-0-mm-3D_firstorder_Uniformity	0.94884469
log-sigma-1-0-mm-3D_firstorder_Variance	0.64009271
log-sigma-1-0-mm-3D_glcm_Autocorrelation	0.40986033
log-sigma-1-0-mm-3D_glcm_JointAverage	0.70711904
log-sigma-1-0-mm-3D_glcm_ClusterProminence	0.12060368
log-sigma-1-0-mm-3D_glcm_ClusterShade	0.15858996
log-sigma-1-0-mm-3D_glcm_ClusterTendency	0.62200113
log-sigma-1-0-mm-3D_glcm_Contrast	0.54827517
log-sigma-1-0-mm-3D_glcm_Correlation	0.91002909
log-sigma-1-0-mm-3D_glcm_DifferenceAverage	0.81065788
log-sigma-1-0-mm-3D_glcm_DifferenceEntropy	0.90021914
log-sigma-1-0-mm-3D_glcm_DifferenceVariance	0.51981831
log-sigma-1-0-mm-3D_glcm_JointEnergy	0.95640591
log-sigma-1-0-mm-3D_glcm_JointEntropy	0.92374199
log-sigma-1-0-mm-3D_glcm_Imc1	0.88140865
log-sigma-1-0-mm-3D_glcm_Imc2	0.92055203
log-sigma-1-0-mm-3D_glcm_Idm	0.92883655
log-sigma-1-0-mm-3D_glcm_Idmn	0.83207559
log-sigma-1-0-mm-3D_glcm_Id	0.9328718
log-sigma-1-0-mm-3D_glcm_Idn	0.89334546
log-sigma-1-0-mm-3D_glcm_InverseVariance	0.98250242
log-sigma-1-0-mm-3D_glcm_MaximumProbability	0.98421738
log-sigma-1-0-mm-3D_glcm_SumEntropy	0.92995609
log-sigma-1-0-mm-3D_glcm_SumSquares	0.6066355
log-sigma-1-0-mm-3D_glrlm_GrayLevelNonUniformity	0.98084857
log-sigma-1-0-mm-3D_glrlm_GrayLevelNonUniformityNormalized	0.94198623
log-sigma-1-0-mm-3D_glrlm_GrayLevelVariance	0.59579821
log-sigma-1-0-mm-3D_glrlm_HighGrayLevelRunEmphasis	0.41213725
log-sigma-1-0-mm-3D_glrlm_LongRunEmphasis	0.97327303
log-sigma-1-0-mm-3D_glrlm_LongRunHighGrayLevelEmphasis	0.31970075

log-sigma-1-0-mm-3D_glrlm_LongRunLowGrayLevelEmphasis	0.68008262
log-sigma-1-0-mm-3D_glrlm_LowGrayLevelRunEmphasis	0.71460671
log-sigma-1-0-mm-3D_glrlm_RunEntropy	0.9222103
log-sigma-1-0-mm-3D_glrlm_RunLengthNonUniformity	0.95863643
log-sigma-1-0-mm-3D_glrlm_RunLengthNonUniformityNormalized	0.95252585
log-sigma-1-0-mm-3D_glrlm_RunPercentage	0.96868718
log-sigma-1-0-mm-3D_glrlm_RunVariance	0.9832015
log-sigma-1-0-mm-3D_glrlm_ShortRunEmphasis	0.95536691
log-sigma-1-0-mm-3D_glrlm_ShortRunHighGrayLevelEmphasis	0.41069701
log-sigma-1-0-mm-3D_glrlm_ShortRunLowGrayLevelEmphasis	0.72217519
log-sigma-1-0-mm-3D_glszm_GrayLevelNonUniformity	0.96557438
log-sigma-1-0-mm-3D_glszm_GrayLevelNonUniformityNormalized	0.92469992
log-sigma-1-0-mm-3D_glszm_GrayLevelVariance	0.70187114
log-sigma-1-0-mm-3D_glszm_HighGrayLevelZoneEmphasis	0.44282444
log-sigma-1-0-mm-3D_glszm_LargeAreaEmphasis	0.73823435
log-sigma-1-0-mm-3D_glszm_LargeAreaHighGrayLevelEmphasis	0.61028389
log-sigma-1-0-mm-3D_glszm_LargeAreaLowGrayLevelEmphasis	0.57186381
log-sigma-1-0-mm-3D_glszm_LowGrayLevelZoneEmphasis	0.63605761
log-sigma-1-0-mm-3D_glszm_SizeZoneNonUniformity	0.8600208
log-sigma-1-0-mm-3D_glszm_SizeZoneNonUniformityNormalized	0.95570439
log-sigma-1-0-mm-3D_glszm_SmallAreaEmphasis	0.94716279
log-sigma-1-0-mm-3D_glszm_SmallAreaHighGrayLevelEmphasis	0.4457688
log-sigma-1-0-mm-3D_glszm_SmallAreaLowGrayLevelEmphasis	0.57620803
log-sigma-1-0-mm-3D_glszm_ZoneEntropy	0.85357476
log-sigma-1-0-mm-3D_glszm_ZonePercentage	0.90192963
log-sigma-1-0-mm-3D_glszm_ZoneVariance	0.73752063
log-sigma-1-0-mm-3D_gldm_DependenceEntropy	0.92434627
log-sigma-1-0-mm-3D_gldm_DependenceNonUniformity	0.97923832
log-sigma-1-0-mm-3D_gldm_DependenceNonUniformityNormalized	0.89604281
log-sigma-1-0-mm-3D_gldm_DependenceVariance	0.90034533
log-sigma-1-0-mm-3D_gldm_GrayLevelNonUniformity	0.97878376
log-sigma-1-0-mm-3D_gldm_GrayLevelVariance	0.63561048
log-sigma-1-0-mm-3D_gldm_HighGrayLevelEmphasis	0.41022847
log-sigma-1-0-mm-3D_gldm_LargeDependenceEmphasis	0.9784045
log-sigma-1-0-mm-3D_gldm_LargeDependenceHighGrayLevelEmphasis	0.38374783
log-sigma-1-0-mm-3D_gldm_LargeDependenceLowGrayLevelEmphasis	0.69948974
log-sigma-1-0-mm-3D_gldm_LowGrayLevelEmphasis	0.71306768
log-sigma-1-0-mm-3D_gldm_SmallDependenceEmphasis	0.91391067
log-sigma-1-0-mm-3D_gldm_SmallDependenceHighGrayLevelEmphasis	0.31247422
log-sigma-1-0-mm-3D_gldm_SmallDependenceLowGrayLevelEmphasis	0.66906497
log-sigma-2-0-mm-3D_firstorder_10Percentile	0.85265506
log-sigma-2-0-mm-3D_firstorder_90Percentile	0.99076849
log-sigma-2-0-mm-3D_firstorder_Energy	0.97594643
log-sigma-2-0-mm-3D_firstorder_Entropy	0.96279304
log-sigma-2-0-mm-3D_firstorder_InterquartileRange	0.95475584
log-sigma-2-0-mm-3D_firstorder_Kurtosis	0.7152522
log-sigma-2-0-mm-3D_firstorder_Maximum	0.83879628
log-sigma-2-0-mm-3D_firstorder_MeanAbsoluteDeviation	0.91313944
log-sigma-2-0-mm-3D_firstorder_Mean	0.86059604

log-sigma-2-0-mm-3D_firstorder_Median	0.9807496
log-sigma-2-0-mm-3D_firstorder_Minimum	0.90839437
log-sigma-2-0-mm-3D_firstorder_Range	0.92209376
log-sigma-2-0-mm-3D_firstorder_RobustMeanAbsoluteDeviation	0.9248078
log-sigma-2-0-mm-3D_firstorder_RootMeanSquared	0.87056136
log-sigma-2-0-mm-3D_firstorder_Skewness	0.75076756
log-sigma-2-0-mm-3D_firstorder_TotalEnergy	0.97594643
log-sigma-2-0-mm-3D_firstorder_Uniformity	0.97538347
log-sigma-2-0-mm-3D_firstorder_Variance	0.8178
log-sigma-2-0-mm-3D_glcm_Autocorrelation	0.84101602
log-sigma-2-0-mm-3D_glcm_JointAverage	0.90941382
log-sigma-2-0-mm-3D_glcm_ClusterProminence	0.26002284
log-sigma-2-0-mm-3D_glcm_ClusterShade	0.42231962
log-sigma-2-0-mm-3D_glcm_ClusterTendency	0.78899588
log-sigma-2-0-mm-3D_glcm_Contrast	0.83870903
log-sigma-2-0-mm-3D_glcm_Correlation	0.96820018
log-sigma-2-0-mm-3D_glcm_DifferenceAverage	0.9262649
log-sigma-2-0-mm-3D_glcm_DifferenceEntropy	0.94837237
log-sigma-2-0-mm-3D_glcm_DifferenceVariance	0.83077826
log-sigma-2-0-mm-3D_glcm_JointEnergy	0.97991889
log-sigma-2-0-mm-3D_glcm_JointEntropy	0.95753378
log-sigma-2-0-mm-3D_glcm_Imc1	0.9598573
log-sigma-2-0-mm-3D_glcm_Imc2	0.97196405
log-sigma-2-0-mm-3D_glcm_Idm	0.95101609
log-sigma-2-0-mm-3D_glcm_Idmn	0.82218801
log-sigma-2-0-mm-3D_glcm_Id	0.95281767
log-sigma-2-0-mm-3D_glcm_Idn	0.81154767
log-sigma-2-0-mm-3D_glcm_InverseVariance	0.9669795
log-sigma-2-0-mm-3D_glcm_MaximumProbability	0.98871626
log-sigma-2-0-mm-3D_glcm_SumEntropy	0.96125092
log-sigma-2-0-mm-3D_glcm_SumSquares	0.79153503
log-sigma-2-0-mm-3D_glrlm_GrayLevelNonUniformity	0.98333088
log-sigma-2-0-mm-3D_glrlm_GrayLevelNonUniformityNormalized	0.95994932
log-sigma-2-0-mm-3D_glrlm_GrayLevelVariance	0.80148449
log-sigma-2-0-mm-3D_glrlm_HighGrayLevelRunEmphasis	0.84174579
log-sigma-2-0-mm-3D_glrlm_LongRunEmphasis	0.97820718
log-sigma-2-0-mm-3D_glrlm_LongRunHighGrayLevelEmphasis	0.84387294
log-sigma-2-0-mm-3D_glrlm_LongRunLowGrayLevelEmphasis	0.92753136
log-sigma-2-0-mm-3D_glrlm_LowGrayLevelRunEmphasis	0.89936139
log-sigma-2-0-mm-3D_glrlm_RunEntropy	0.952662
log-sigma-2-0-mm-3D_glrlm_RunLengthNonUniformity	0.94116887
log-sigma-2-0-mm-3D_glrlm_RunLengthNonUniformityNormalized	0.94859912
log-sigma-2-0-mm-3D_glrlm_RunPercentage	0.96682822
log-sigma-2-0-mm-3D_glrlm_RunVariance	0.98589773
log-sigma-2-0-mm-3D_glrlm_ShortRunEmphasis	0.9507957
log-sigma-2-0-mm-3D_glrlm_ShortRunHighGrayLevelEmphasis	0.82230589
log-sigma-2-0-mm-3D_glrlm_ShortRunLowGrayLevelEmphasis	0.85500173
log-sigma-2-0-mm-3D_glszm_GrayLevelNonUniformity	0.94575062
log-sigma-2-0-mm-3D_glszm_GrayLevelNonUniformityNormalized	0.93360024

log-sigma-2-0-mm-3D_glszm_GrayLevelVariance	0.76512091
log-sigma-2-0-mm-3D_glszm_HighGrayLevelZoneEmphasis	0.84844464
log-sigma-2-0-mm-3D_glszm_LargeAreaEmphasis	0.78747006
log-sigma-2-0-mm-3D_glszm_LargeAreaHighGrayLevelEmphasis	0.73623995
log-sigma-2-0-mm-3D_glszm_LargeAreaLowGrayLevelEmphasis	0.86978247
log-sigma-2-0-mm-3D_glszm_LowGrayLevelZoneEmphasis	0.87785816
log-sigma-2-0-mm-3D_glszm_SizeZoneNonUniformity	0.96738913
log-sigma-2-0-mm-3D_glszm_SizeZoneNonUniformityNormalized	0.95233341
log-sigma-2-0-mm-3D_glszm_SmallAreaEmphasis	0.93658604
log-sigma-2-0-mm-3D_glszm_SmallAreaHighGrayLevelEmphasis	0.86682145
log-sigma-2-0-mm-3D_glszm_SmallAreaLowGrayLevelEmphasis	0.8038756
log-sigma-2-0-mm-3D_glszm_ZoneEntropy	0.72984209
log-sigma-2-0-mm-3D_glszm_ZonePercentage	0.96875229
log-sigma-2-0-mm-3D_glszm_ZoneVariance	0.78672266
log-sigma-2-0-mm-3D_gldm_DependenceEntropy	0.97315756
log-sigma-2-0-mm-3D_gldm_DependenceNonUniformity	0.98291955
log-sigma-2-0-mm-3D_gldm_DependenceNonUniformityNormalized	0.98744726
log-sigma-2-0-mm-3D_gldm_DependenceVariance	0.94435914
log-sigma-2-0-mm-3D_gldm_GrayLevelNonUniformity	0.98413613
log-sigma-2-0-mm-3D_gldm_GrayLevelVariance	0.81871302
log-sigma-2-0-mm-3D_gldm_HighGrayLevelEmphasis	0.84041786
log-sigma-2-0-mm-3D_gldm_LargeDependenceEmphasis	0.97156638
log-sigma-2-0-mm-3D_gldm_LargeDependenceHighGrayLevelEmphasis	0.85131615
log-sigma-2-0-mm-3D_gldm_LargeDependenceLowGrayLevelEmphasis	0.92819036
log-sigma-2-0-mm-3D_gldm_LowGrayLevelEmphasis	0.91359476
log-sigma-2-0-mm-3D_gldm_SmallDependenceEmphasis	0.96782557
log-sigma-2-0-mm-3D_gldm_SmallDependenceHighGrayLevelEmphasis	0.78748009
log-sigma-2-0-mm-3D_gldm_SmallDependenceLowGrayLevelEmphasis	0.80180529
log-sigma-3-0-mm-3D_firstorder_10Percentile	0.94664604
log-sigma-3-0-mm-3D_firstorder_90Percentile	0.98125323
log-sigma-3-0-mm-3D_firstorder_Energy	0.97642051
log-sigma-3-0-mm-3D_firstorder_Entropy	0.97497064
log-sigma-3-0-mm-3D_firstorder_InterquartileRange	0.96003667
log-sigma-3-0-mm-3D_firstorder_Kurtosis	0.9556044
log-sigma-3-0-mm-3D_firstorder_Maximum	0.65480767
log-sigma-3-0-mm-3D_firstorder_MeanAbsoluteDeviation	0.9590122
log-sigma-3-0-mm-3D_firstorder_Mean	0.94389275
log-sigma-3-0-mm-3D_firstorder_Median	0.98464001
log-sigma-3-0-mm-3D_firstorder_Minimum	0.96448204
log-sigma-3-0-mm-3D_firstorder_Range	0.93801616
log-sigma-3-0-mm-3D_firstorder_RobustMeanAbsoluteDeviation	0.93912623
log-sigma-3-0-mm-3D_firstorder_RootMeanSquared	0.94486196
log-sigma-3-0-mm-3D_firstorder_Skewness	0.91619445
log-sigma-3-0-mm-3D_firstorder_TotalEnergy	0.97642051
log-sigma-3-0-mm-3D_firstorder_Uniformity	0.98169318
log-sigma-3-0-mm-3D_firstorder_Variance	0.94132509
log-sigma-3-0-mm-3D_glcm_Autocorrelation	0.96446594
log-sigma-3-0-mm-3D_glcm_JointAverage	0.96767067
log-sigma-3-0-mm-3D_glcm_ClusterProminence	0.63246383

log-sigma-3-0-mm-3D_glcm_ClusterShade	0.79356982
log-sigma-3-0-mm-3D_glcm_ClusterTendency	0.92396877
log-sigma-3-0-mm-3D_glcm_Contrast	0.95115118
log-sigma-3-0-mm-3D_glcm_Correlation	0.98907907
log-sigma-3-0-mm-3D_glcm_DifferenceAverage	0.96375729
log-sigma-3-0-mm-3D_glcm_DifferenceEntropy	0.9679238
log-sigma-3-0-mm-3D_glcm_DifferenceVariance	0.95242916
log-sigma-3-0-mm-3D_glcm_JointEnergy	0.98375066
log-sigma-3-0-mm-3D_glcm_JointEntropy	0.97147158
log-sigma-3-0-mm-3D_glcm_Imc1	0.95751762
log-sigma-3-0-mm-3D_glcm_Imc2	0.98918051
log-sigma-3-0-mm-3D_glcm_Idm	0.96781021
log-sigma-3-0-mm-3D_glcm_Idmn	0.63884861
log-sigma-3-0-mm-3D_glcm_Id	0.96832071
log-sigma-3-0-mm-3D_glcm_Idn	0.7452438
log-sigma-3-0-mm-3D_glcm_InverseVariance	0.97269846
log-sigma-3-0-mm-3D_glcm_MaximumProbability	0.98879525
log-sigma-3-0-mm-3D_glcm_SumEntropy	0.97402756
log-sigma-3-0-mm-3D_glcm_SumSquares	0.92502032
log-sigma-3-0-mm-3D_glrlm_GrayLevelNonUniformity	0.98323446
log-sigma-3-0-mm-3D_glrlm_GrayLevelNonUniformityNormalized	0.98126963
log-sigma-3-0-mm-3D_glrlm_GrayLevelVariance	0.94426366
log-sigma-3-0-mm-3D_glrlm_HighGrayLevelRunEmphasis	0.96435933
log-sigma-3-0-mm-3D_glrlm_LongRunEmphasis	0.98347582
log-sigma-3-0-mm-3D_glrlm_LongRunHighGrayLevelEmphasis	0.94657151
log-sigma-3-0-mm-3D_glrlm_LongRunLowGrayLevelEmphasis	0.95745477
log-sigma-3-0-mm-3D_glrlm_LowGrayLevelRunEmphasis	0.89715825
log-sigma-3-0-mm-3D_glrlm_RunEntropy	0.97746358
log-sigma-3-0-mm-3D_glrlm_RunLengthNonUniformity	0.95086046
log-sigma-3-0-mm-3D_glrlm_RunLengthNonUniformityNormalized	0.96635706
log-sigma-3-0-mm-3D_glrlm_RunPercentage	0.97549636
log-sigma-3-0-mm-3D_glrlm_RunVariance	0.9874003
log-sigma-3-0-mm-3D_glrlm_ShortRunEmphasis	0.9677548
log-sigma-3-0-mm-3D_glrlm_ShortRunHighGrayLevelEmphasis	0.96207323
log-sigma-3-0-mm-3D_glrlm_ShortRunLowGrayLevelEmphasis	0.86577101
log-sigma-3-0-mm-3D_glszm_GrayLevelNonUniformity	0.91731199
log-sigma-3-0-mm-3D_glszm_GrayLevelNonUniformityNormalized	0.96597915
log-sigma-3-0-mm-3D_glszm_GrayLevelVariance	0.9014553
log-sigma-3-0-mm-3D_glszm_HighGrayLevelZoneEmphasis	0.95294892
log-sigma-3-0-mm-3D_glszm_LargeAreaEmphasis	0.83124226
log-sigma-3-0-mm-3D_glszm_LargeAreaHighGrayLevelEmphasis	0.8817423
log-sigma-3-0-mm-3D_glszm_LargeAreaLowGrayLevelEmphasis	0.8852987
log-sigma-3-0-mm-3D_glszm_LowGrayLevelZoneEmphasis	0.85843143
log-sigma-3-0-mm-3D_glszm_SizeZoneNonUniformity	0.96799077
log-sigma-3-0-mm-3D_glszm_SizeZoneNonUniformityNormalized	0.92995344
log-sigma-3-0-mm-3D_glszm_SmallAreaEmphasis	0.91104463
log-sigma-3-0-mm-3D_glszm_SmallAreaHighGrayLevelEmphasis	0.96335588
log-sigma-3-0-mm-3D_glszm_SmallAreaLowGrayLevelEmphasis	0.71707378
log-sigma-3-0-mm-3D_glszm_ZoneEntropy	0.82528472

log-sigma-3-0-mm-3D_glszm_ZonePercentage	0.97119135
log-sigma-3-0-mm-3D_glszm_ZoneVariance	0.8304456
log-sigma-3-0-mm-3D_gldm_DependenceEntropy	0.98016599
log-sigma-3-0-mm-3D_gldm_DependenceNonUniformity	0.98747787
log-sigma-3-0-mm-3D_gldm_DependenceNonUniformityNormalized	0.98678381
log-sigma-3-0-mm-3D_gldm_DependenceVariance	0.96999587
log-sigma-3-0-mm-3D_gldm_GrayLevelNonUniformity	0.98576955
log-sigma-3-0-mm-3D_gldm_GrayLevelVariance	0.94213754
log-sigma-3-0-mm-3D_gldm_HighGrayLevelEmphasis	0.96394776
log-sigma-3-0-mm-3D_gldm_LargeDependenceEmphasis	0.97645999
log-sigma-3-0-mm-3D_gldm_LargeDependenceHighGrayLevelEmphasis	0.96482391
log-sigma-3-0-mm-3D_gldm_LargeDependenceLowGrayLevelEmphasis	0.94644856
log-sigma-3-0-mm-3D_gldm_LowGrayLevelEmphasis	0.92094702
log-sigma-3-0-mm-3D_gldm_SmallDependenceEmphasis	0.97721671
log-sigma-3-0-mm-3D_gldm_SmallDependenceHighGrayLevelEmphasis	0.96527535
log-sigma-3-0-mm-3D_gldm_SmallDependenceLowGrayLevelEmphasis	0.78144419
log-sigma-4-0-mm-3D_firstorder_10Percentile	0.97540343
log-sigma-4-0-mm-3D_firstorder_90Percentile	0.94673084
log-sigma-4-0-mm-3D_firstorder_Energy	0.97663221
log-sigma-4-0-mm-3D_firstorder_Entropy	0.98144451
log-sigma-4-0-mm-3D_firstorder_InterquartileRange	0.95144713
log-sigma-4-0-mm-3D_firstorder_Kurtosis	0.96637154
log-sigma-4-0-mm-3D_firstorder_Maximum	0.44095988
log-sigma-4-0-mm-3D_firstorder_MeanAbsoluteDeviation	0.9778972
log-sigma-4-0-mm-3D_firstorder_Mean	0.96985102
log-sigma-4-0-mm-3D_firstorder_Median	0.97801392
log-sigma-4-0-mm-3D_firstorder_Minimum	0.99604032
log-sigma-4-0-mm-3D_firstorder_Range	0.90581404
log-sigma-4-0-mm-3D_firstorder_RobustMeanAbsoluteDeviation	0.96217821
log-sigma-4-0-mm-3D_firstorder_RootMeanSquared	0.96936834
log-sigma-4-0-mm-3D_firstorder_Skewness	0.93531552
log-sigma-4-0-mm-3D_firstorder_TotalEnergy	0.97663221
log-sigma-4-0-mm-3D_firstorder_Uniformity	0.98402216
log-sigma-4-0-mm-3D_firstorder_Variance	0.97819973
log-sigma-4-0-mm-3D_glcm_Autocorrelation	0.99541581
log-sigma-4-0-mm-3D_glcm_JointAverage	0.99654414
log-sigma-4-0-mm-3D_glcm_ClusterProminence	0.92391539
log-sigma-4-0-mm-3D_glcm_ClusterShade	0.95844284
log-sigma-4-0-mm-3D_glcm_ClusterTendency	0.97186246
log-sigma-4-0-mm-3D_glcm_Contrast	0.96552656
log-sigma-4-0-mm-3D_glcm_Correlation	0.99168019
log-sigma-4-0-mm-3D_glcm_DifferenceAverage	0.97306258
log-sigma-4-0-mm-3D_glcm_DifferenceEntropy	0.97267721
log-sigma-4-0-mm-3D_glcm_DifferenceVariance	0.96351043
log-sigma-4-0-mm-3D_glcm_JointEnergy	0.98435625
log-sigma-4-0-mm-3D_glcm_JointEntropy	0.97791229
log-sigma-4-0-mm-3D_glcm_Imc1	0.95353565
log-sigma-4-0-mm-3D_glcm_Imc2	0.99520399
log-sigma-4-0-mm-3D_glcm_Idm	0.97537167

log-sigma-4-0-mm-3D_glcm_Idmn	0.61115609
log-sigma-4-0-mm-3D_glcm_Id	0.97580812
log-sigma-4-0-mm-3D_glcm_Idn	0.79324929
log-sigma-4-0-mm-3D_glcm_InverseVariance	0.97899934
log-sigma-4-0-mm-3D_glcm_MaximumProbability	0.98728536
log-sigma-4-0-mm-3D_glcm_SumEntropy	0.98043248
log-sigma-4-0-mm-3D_glcm_SumSquares	0.97196578
log-sigma-4-0-mm-3D_glrlm_GrayLevelNonUniformity	0.97769861
log-sigma-4-0-mm-3D_glrlm_GrayLevelNonUniformityNormalized	0.98942361
log-sigma-4-0-mm-3D_glrlm_GrayLevelVariance	0.98745153
log-sigma-4-0-mm-3D_glrlm_HighGrayLevelRunEmphasis	0.99458261
log-sigma-4-0-mm-3D_glrlm_LongRunEmphasis	0.98455155
log-sigma-4-0-mm-3D_glrlm_LongRunHighGrayLevelEmphasis	0.99387726
log-sigma-4-0-mm-3D_glrlm_LongRunLowGrayLevelEmphasis	0.95629874
log-sigma-4-0-mm-3D_glrlm_LowGrayLevelRunEmphasis	0.97964771
log-sigma-4-0-mm-3D_glrlm_RunEntropy	0.98664338
log-sigma-4-0-mm-3D_glrlm_RunLengthNonUniformity	0.95833505
log-sigma-4-0-mm-3D_glrlm_RunLengthNonUniformityNormalized	0.97773514
log-sigma-4-0-mm-3D_glrlm_RunPercentage	0.9811887
log-sigma-4-0-mm-3D_glrlm_RunVariance	0.98656515
log-sigma-4-0-mm-3D_glrlm_ShortRunEmphasis	0.97745057
log-sigma-4-0-mm-3D_glrlm_ShortRunHighGrayLevelEmphasis	0.99317826
log-sigma-4-0-mm-3D_glrlm_ShortRunLowGrayLevelEmphasis	0.98434497
log-sigma-4-0-mm-3D_glszm_GrayLevelNonUniformity	0.9146067
log-sigma-4-0-mm-3D_glszm_GrayLevelNonUniformityNormalized	0.9498475
log-sigma-4-0-mm-3D_glszm_GrayLevelVariance	0.96776368
log-sigma-4-0-mm-3D_glszm_HighGrayLevelZoneEmphasis	0.977889
log-sigma-4-0-mm-3D_glszm_LargeAreaEmphasis	0.85166935
log-sigma-4-0-mm-3D_glszm_LargeAreaHighGrayLevelEmphasis	0.8844069
log-sigma-4-0-mm-3D_glszm_LargeAreaLowGrayLevelEmphasis	0.86954392
log-sigma-4-0-mm-3D_glszm_LowGrayLevelZoneEmphasis	0.94210794
log-sigma-4-0-mm-3D_glszm_SizeZoneNonUniformity	0.95418516
log-sigma-4-0-mm-3D_glszm_SizeZoneNonUniformityNormalized	0.84361828
log-sigma-4-0-mm-3D_glszm_SmallAreaEmphasis	0.83401082
log-sigma-4-0-mm-3D_glszm_SmallAreaHighGrayLevelEmphasis	0.98366968
log-sigma-4-0-mm-3D_glszm_SmallAreaLowGrayLevelEmphasis	0.77838983
log-sigma-4-0-mm-3D_glszm_ZoneEntropy	0.78103769
log-sigma-4-0-mm-3D_glszm_ZonePercentage	0.95144326
log-sigma-4-0-mm-3D_glszm_ZoneVariance	0.85057969
log-sigma-4-0-mm-3D_gldm_DependenceEntropy	0.98137293
log-sigma-4-0-mm-3D_gldm_DependenceNonUniformity	0.98534586
log-sigma-4-0-mm-3D_gldm_DependenceNonUniformityNormalized	0.98335735
log-sigma-4-0-mm-3D_gldm_DependenceVariance	0.97400715
log-sigma-4-0-mm-3D_gldm_GrayLevelNonUniformity	0.98306587
log-sigma-4-0-mm-3D_gldm_GrayLevelVariance	0.97840191
log-sigma-4-0-mm-3D_gldm_HighGrayLevelEmphasis	0.9951674
log-sigma-4-0-mm-3D_gldm_LargeDependenceEmphasis	0.98140136
log-sigma-4-0-mm-3D_gldm_LargeDependenceHighGrayLevelEmphasis	0.99713597
log-sigma-4-0-mm-3D_gldm_LargeDependenceLowGrayLevelEmphasis	0.96993714

log-sigma-4-0-mm-3D_gldm_LowGrayLevelEmphasis	0.97474735
log-sigma-4-0-mm-3D_gldm_SmallDependenceEmphasis	0.97242017
log-sigma-4-0-mm-3D_gldm_SmallDependenceHighGrayLevelEmphasis	0.99104164
log-sigma-4-0-mm-3D_gldm_SmallDependenceLowGrayLevelEmphasis	0.96845856
log-sigma-5-0-mm-3D_firstorder_10Percentile	0.98505815
log-sigma-5-0-mm-3D_firstorder_90Percentile	0.96235262
log-sigma-5-0-mm-3D_firstorder_Energy	0.97665281
log-sigma-5-0-mm-3D_firstorder_Entropy	0.98589372
log-sigma-5-0-mm-3D_firstorder_InterquartileRange	0.97149664
log-sigma-5-0-mm-3D_firstorder_Kurtosis	0.97534142
log-sigma-5-0-mm-3D_firstorder_Maximum	0.41889412
log-sigma-5-0-mm-3D_firstorder_MeanAbsoluteDeviation	0.98612146
log-sigma-5-0-mm-3D_firstorder_Mean	0.97964809
log-sigma-5-0-mm-3D_firstorder_Median	0.97483229
log-sigma-5-0-mm-3D_firstorder_Minimum	0.99699885
log-sigma-5-0-mm-3D_firstorder_Range	0.84564968
log-sigma-5-0-mm-3D_firstorder_RobustMeanAbsoluteDeviation	0.97554193
log-sigma-5-0-mm-3D_firstorder_RootMeanSquared	0.97902643
log-sigma-5-0-mm-3D_firstorder_Skewness	0.9515939
log-sigma-5-0-mm-3D_firstorder_TotalEnergy	0.97665281
log-sigma-5-0-mm-3D_firstorder_Uniformity	0.98536554
log-sigma-5-0-mm-3D_firstorder_Variance	0.98790497
log-sigma-5-0-mm-3D_glcm_Autocorrelation	0.98910335
log-sigma-5-0-mm-3D_glcm_JointAverage	0.99314175
log-sigma-5-0-mm-3D_glcm_ClusterProminence	0.9912105
log-sigma-5-0-mm-3D_glcm_ClusterShade	0.9944154
log-sigma-5-0-mm-3D_glcm_ClusterTendency	0.98581442
log-sigma-5-0-mm-3D_glcm_Contrast	0.96807527
log-sigma-5-0-mm-3D_glcm_Correlation	0.9894203
log-sigma-5-0-mm-3D_glcm_DifferenceAverage	0.97690329
log-sigma-5-0-mm-3D_glcm_DifferenceEntropy	0.9744777
log-sigma-5-0-mm-3D_glcm_DifferenceVariance	0.96323127
log-sigma-5-0-mm-3D_glcm_JointEnergy	0.98452602
log-sigma-5-0-mm-3D_glcm_JointEntropy	0.98246227
log-sigma-5-0-mm-3D_glcm_Imc1	0.94757624
log-sigma-5-0-mm-3D_glcm_Imc2	0.99625629
log-sigma-5-0-mm-3D_glcm_Idm	0.97932036
log-sigma-5-0-mm-3D_glcm_Idmn	0.55265413
log-sigma-5-0-mm-3D_glcm_Id	0.98006349
log-sigma-5-0-mm-3D_glcm_Idn	0.8306616
log-sigma-5-0-mm-3D_glcm_InverseVariance	0.98400165
log-sigma-5-0-mm-3D_glcm_MaximumProbability	0.98704202
log-sigma-5-0-mm-3D_glcm_SumEntropy	0.98505424
log-sigma-5-0-mm-3D_glcm_SumSquares	0.98576912
log-sigma-5-0-mm-3D_glrlm_GrayLevelNonUniformity	0.97071817
log-sigma-5-0-mm-3D_glrlm_GrayLevelNonUniformityNormalized	0.99370256
log-sigma-5-0-mm-3D_glrlm_GrayLevelVariance	0.99519725
log-sigma-5-0-mm-3D_glrlm_HighGrayLevelRunEmphasis	0.98591573
log-sigma-5-0-mm-3D_glrlm_LongRunEmphasis	0.98411613

log-sigma-5-0-mm-3D_glrlm_LongRunHighGrayLevelEmphasis	0.97382145
log-sigma-5-0-mm-3D_glrlm_LongRunLowGrayLevelEmphasis	0.99425522
log-sigma-5-0-mm-3D_glrlm_LowGrayLevelRunEmphasis	0.99422102
log-sigma-5-0-mm-3D_glrlm_RunEntropy	0.9880374
log-sigma-5-0-mm-3D_glrlm_RunLengthNonUniformity	0.96052295
log-sigma-5-0-mm-3D_glrlm_RunLengthNonUniformityNormalized	0.98154194
log-sigma-5-0-mm-3D_glrlm_RunPercentage	0.98468344
log-sigma-5-0-mm-3D_glrlm_RunVariance	0.98377772
log-sigma-5-0-mm-3D_glrlm_ShortRunEmphasis	0.97925422
log-sigma-5-0-mm-3D_glrlm_ShortRunHighGrayLevelEmphasis	0.98625245
log-sigma-5-0-mm-3D_glrlm_ShortRunLowGrayLevelEmphasis	0.99145908
log-sigma-5-0-mm-3D_glszm_GrayLevelNonUniformity	0.92707333
log-sigma-5-0-mm-3D_glszm_GrayLevelNonUniformityNormalized	0.89978548
log-sigma-5-0-mm-3D_glszm_GrayLevelVariance	0.92820422
log-sigma-5-0-mm-3D_glszm_HighGrayLevelZoneEmphasis	0.93743701
log-sigma-5-0-mm-3D_glszm_LargeAreaEmphasis	0.88018503
log-sigma-5-0-mm-3D_glszm_LargeAreaHighGrayLevelEmphasis	0.89966822
log-sigma-5-0-mm-3D_glszm_LargeAreaLowGrayLevelEmphasis	0.89544265
log-sigma-5-0-mm-3D_glszm_LowGrayLevelZoneEmphasis	0.92845222
log-sigma-5-0-mm-3D_glszm_SizeZoneNonUniformity	0.94637582
log-sigma-5-0-mm-3D_glszm_SizeZoneNonUniformityNormalized	0.76765541
log-sigma-5-0-mm-3D_glszm_SmallAreaEmphasis	0.76159143
log-sigma-5-0-mm-3D_glszm_SmallAreaHighGrayLevelEmphasis	0.96661442
log-sigma-5-0-mm-3D_glszm_SmallAreaLowGrayLevelEmphasis	0.70326764
log-sigma-5-0-mm-3D_glszm_ZoneEntropy	0.64235288
log-sigma-5-0-mm-3D_glszm_ZonePercentage	0.94482518
log-sigma-5-0-mm-3D_glszm_ZoneVariance	0.87880038
log-sigma-5-0-mm-3D_gldm_DependenceEntropy	0.9834906
log-sigma-5-0-mm-3D_gldm_DependenceNonUniformity	0.98332587
log-sigma-5-0-mm-3D_gldm_DependenceNonUniformityNormalized	0.98334314
log-sigma-5-0-mm-3D_gldm_DependenceVariance	0.96779254
log-sigma-5-0-mm-3D_gldm_GrayLevelNonUniformity	0.98053
log-sigma-5-0-mm-3D_gldm_GrayLevelVariance	0.98794588
log-sigma-5-0-mm-3D_gldm_HighGrayLevelEmphasis	0.98867888
log-sigma-5-0-mm-3D_gldm_LargeDependenceEmphasis	0.98539029
log-sigma-5-0-mm-3D_gldm_LargeDependenceHighGrayLevelEmphasis	0.989826
log-sigma-5-0-mm-3D_gldm_LargeDependenceLowGrayLevelEmphasis	0.99826321
log-sigma-5-0-mm-3D_gldm_LowGrayLevelEmphasis	0.99558598
log-sigma-5-0-mm-3D_gldm_SmallDependenceEmphasis	0.97220227
log-sigma-5-0-mm-3D_gldm_SmallDependenceHighGrayLevelEmphasis	0.98425227
log-sigma-5-0-mm-3D_gldm_SmallDependenceLowGrayLevelEmphasis	0.97856508
wavelet-LLH_firstorder_10Percentile	0.99281861
wavelet-LLH_firstorder_90Percentile	0.99751178
wavelet-LLH_firstorder_Energy	0.97434545
wavelet-LLH_firstorder_Entropy	0.9830908
wavelet-LLH_firstorder_InterquartileRange	0.99878165
wavelet-LLH_firstorder_Kurtosis	0.98874713
wavelet-LLH_firstorder_Maximum	0.97967103
wavelet-LLH_firstorder_MeanAbsoluteDeviation	0.99030413

wavelet-LLH_firstorder_Mean	0.80848911
wavelet-LLH_firstorder_Median	0.9499557
wavelet-LLH_firstorder_Minimum	0.99393128
wavelet-LLH_firstorder_Range	0.99444441
wavelet-LLH_firstorder_RobustMeanAbsoluteDeviation	0.99834332
wavelet-LLH_firstorder_RootMeanSquared	0.62900378
wavelet-LLH_firstorder_Skewness	0.73806388
wavelet-LLH_firstorder_TotalEnergy	0.97434545
wavelet-LLH_firstorder_Uniformity	0.9856335
wavelet-LLH_firstorder_Variance	0.95982043
wavelet-LLH_glcm_Autocorrelation	0.99889572
wavelet-LLH_glcm_JointAverage	0.99274336
wavelet-LLH_glcm_ClusterProminence	0.80695972
wavelet-LLH_glcm_ClusterShade	0.88637728
wavelet-LLH_glcm_ClusterTendency	0.93984193
wavelet-LLH_glcm_Contrast	0.93842935
wavelet-LLH_glcm_Correlation	0.93479644
wavelet-LLH_glcm_DifferenceAverage	0.9883731
wavelet-LLH_glcm_DifferenceEntropy	0.99063341
wavelet-LLH_glcm_DifferenceVariance	0.92620981
wavelet-LLH_glcm_JointEnergy	0.99045904
wavelet-LLH_glcm_JointEntropy	0.98469793
wavelet-LLH_glcm_Imc1	0.92806692
wavelet-LLH_glcm_Imc2	0.88774008
wavelet-LLH_glcm_Idm	0.99593654
wavelet-LLH_glcm_Idmn	0.96333906
wavelet-LLH_glcm_Id	0.99621919
wavelet-LLH_glcm_Idn	0.96564754
wavelet-LLH_glcm_InverseVariance	0.99614095
wavelet-LLH_glcm_MaximumProbability	0.98917583
wavelet-LLH_glcm_SumEntropy	0.97182171
wavelet-LLH_glcm_SumSquares	0.93946373
wavelet-LLH_glrlm_GrayLevelNonUniformity	0.97788493
wavelet-LLH_glrlm_GrayLevelNonUniformityNormalized	0.98008526
wavelet-LLH_glrlm_GrayLevelVariance	0.96098074
wavelet-LLH_glrlm_HighGrayLevelRunEmphasis	0.99890395
wavelet-LLH_glrlm_LongRunEmphasis	0.99015138
wavelet-LLH_glrlm_LongRunHighGrayLevelEmphasis	0.99042483
wavelet-LLH_glrlm_LongRunLowGrayLevelEmphasis	0.95575347
wavelet-LLH_glrlm_LowGrayLevelRunEmphasis	0.96306299
wavelet-LLH_glrlm_RunEntropy	0.899703
wavelet-LLH_glrlm_RunLengthNonUniformity	0.9825283
wavelet-LLH_glrlm_RunLengthNonUniformityNormalized	0.99790967
wavelet-LLH_glrlm_RunPercentage	0.99766699
wavelet-LLH_glrlm_RunVariance	0.9848878
wavelet-LLH_glrlm_ShortRunEmphasis	0.99710816
wavelet-LLH_glrlm_ShortRunHighGrayLevelEmphasis	0.99941195
wavelet-LLH_glrlm_ShortRunLowGrayLevelEmphasis	0.96266016
wavelet-LLH_glszm_GrayLevelNonUniformity	0.99778791

wavelet-LLH_glszm_GrayLevelNonUniformityNormalized	0.95486722
wavelet-LLH_glszm_GrayLevelVariance	0.97431282
wavelet-LLH_glszm_HighGrayLevelZoneEmphasis	0.99908741
wavelet-LLH_glszm_LargeAreaEmphasis	0.88015862
wavelet-LLH_glszm_LargeAreaHighGrayLevelEmphasis	0.98139417
wavelet-LLH_glszm_LargeAreaLowGrayLevelEmphasis	0.73455707
wavelet-LLH_glszm_LowGrayLevelZoneEmphasis	0.86893649
wavelet-LLH_glszm_SizeZoneNonUniformity	0.98142646
wavelet-LLH_glszm_SizeZoneNonUniformityNormalized	0.75686849
wavelet-LLH_glszm_SmallAreaEmphasis	0.74450822
wavelet-LLH_glszm_SmallAreaHighGrayLevelEmphasis	0.99969761
wavelet-LLH_glszm_SmallAreaLowGrayLevelEmphasis	0.88966319
wavelet-LLH_glszm_ZoneEntropy	0.91486329
wavelet-LLH_glszm_ZonePercentage	0.99579401
wavelet-LLH_glszm_ZoneVariance	0.87933333
wavelet-LLH_gldm_DependenceEntropy	0.91334054
wavelet-LLH_gldm_DependenceNonUniformity	0.97676758
wavelet-LLH_gldm_DependenceNonUniformityNormalized	0.98653275
wavelet-LLH_gldm_DependenceVariance	0.9690586
wavelet-LLH_gldm_GrayLevelNonUniformity	0.97712151
wavelet-LLH_gldm_GrayLevelVariance	0.95530817
wavelet-LLH_gldm_HighGrayLevelEmphasis	0.99889902
wavelet-LLH_gldm_LargeDependenceEmphasis	0.99699738
wavelet-LLH_gldm_LargeDependenceHighGrayLevelEmphasis	0.99550645
wavelet-LLH_gldm_LargeDependenceLowGrayLevelEmphasis	0.96187095
wavelet-LLH_gldm_LowGrayLevelEmphasis	0.96417635
wavelet-LLH_gldm_SmallDependenceEmphasis	0.99607226
wavelet-LLH_gldm_SmallDependenceHighGrayLevelEmphasis	0.99662921
wavelet-LLH_gldm_SmallDependenceLowGrayLevelEmphasis	0.95975426
wavelet-LHL_firstorder_10Percentile	0.93818946
wavelet-LHL_firstorder_90Percentile	0.96008198
wavelet-LHL_firstorder_Energy	0.97424558
wavelet-LHL_firstorder_Entropy	0.93123276
wavelet-LHL_firstorder_InterquartileRange	0.98009109
wavelet-LHL_firstorder_Kurtosis	0.95765687
wavelet-LHL_firstorder_Maximum	0.96118026
wavelet-LHL_firstorder_MeanAbsoluteDeviation	0.84114511
wavelet-LHL_firstorder_Mean	0.61081966
wavelet-LHL_firstorder_Median	0.95871154
wavelet-LHL_firstorder_Minimum	0.98479663
wavelet-LHL_firstorder_Range	0.9812827
wavelet-LHL_firstorder_RobustMeanAbsoluteDeviation	0.9732979
wavelet-LHL_firstorder_RootMeanSquared	0.94219818
wavelet-LHL_firstorder_Skewness	0.92822956
wavelet-LHL_firstorder_TotalEnergy	0.97424558
wavelet-LHL_firstorder_Uniformity	0.95868516
wavelet-LHL_firstorder_Variance	0.63357657
wavelet-LHL_glcm_Autocorrelation	0.98086408
wavelet-LHL_glcm_JointAverage	0.98577468

wavelet-LHL_glcm_ClusterProminence	0.23710651
wavelet-LHL_glcm_ClusterShade	0.14429665
wavelet-LHL_glcm_ClusterTendency	0.5487561
wavelet-LHL_glcm_Contrast	0.56368488
wavelet-LHL_glcm_Correlation	0.95843715
wavelet-LHL_glcm_DifferenceAverage	0.81776175
wavelet-LHL_glcm_DifferenceEntropy	0.92470713
wavelet-LHL_glcm_DifferenceVariance	0.55183418
wavelet-LHL_glcm_JointEnergy	0.97214917
wavelet-LHL_glcm_JointEntropy	0.92804509
wavelet-LHL_glcm_Imc1	0.84532862
wavelet-LHL_glcm_Imc2	0.8221125
wavelet-LHL_glcm_Idm	0.96663735
wavelet-LHL_glcm_Idmn	0.91141408
wavelet-LHL_glcm_Id	0.96458226
wavelet-LHL_glcm_Idn	0.9188289
wavelet-LHL_glcm_InverseVariance	0.89597271
wavelet-LHL_glcm_MaximumProbability	0.98005543
wavelet-LHL_glcm_SumEntropy	0.91845356
wavelet-LHL_glcm_SumSquares	0.55308338
wavelet-LHL_glrlm_GrayLevelNonUniformity	0.97582558
wavelet-LHL_glrlm_GrayLevelNonUniformityNormalized	0.94952661
wavelet-LHL_glrlm_GrayLevelVariance	0.63102759
wavelet-LHL_glrlm_HighGrayLevelRunEmphasis	0.98095303
wavelet-LHL_glrlm_LongRunEmphasis	0.9831598
wavelet-LHL_glrlm_LongRunHighGrayLevelEmphasis	0.98538458
wavelet-LHL_glrlm_LongRunLowGrayLevelEmphasis	0.32910509
wavelet-LHL_glrlm_LowGrayLevelRunEmphasis	0.54135946
wavelet-LHL_glrlm_RunEntropy	0.91683659
wavelet-LHL_glrlm_RunLengthNonUniformity	0.96446517
wavelet-LHL_glrlm_RunLengthNonUniformityNormalized	0.98100485
wavelet-LHL_glrlm_RunPercentage	0.98538942
wavelet-LHL_glrlm_RunVariance	0.99000651
wavelet-LHL_glrlm_ShortRunEmphasis	0.97848624
wavelet-LHL_glrlm_ShortRunHighGrayLevelEmphasis	0.97748459
wavelet-LHL_glrlm_ShortRunLowGrayLevelEmphasis	0.59684204
wavelet-LHL_glszm_GrayLevelNonUniformity	0.98432716
wavelet-LHL_glszm_GrayLevelNonUniformityNormalized	0.95394436
wavelet-LHL_glszm_GrayLevelVariance	0.76123406
wavelet-LHL_glszm_HighGrayLevelZoneEmphasis	0.98338519
wavelet-LHL_glszm_LargeAreaEmphasis	0.89682576
wavelet-LHL_glszm_LargeAreaHighGrayLevelEmphasis	0.94604042
wavelet-LHL_glszm_LargeAreaLowGrayLevelEmphasis	0.21164776
wavelet-LHL_glszm_LowGrayLevelZoneEmphasis	0.50438708
wavelet-LHL_glszm_SizeZoneNonUniformity	0.9302501
wavelet-LHL_glszm_SizeZoneNonUniformityNormalized	0.98868939
wavelet-LHL_glszm_SmallAreaEmphasis	0.98776747
wavelet-LHL_glszm_SmallAreaHighGrayLevelEmphasis	0.97634363
wavelet-LHL_glszm_SmallAreaLowGrayLevelEmphasis	0.55100137

wavelet-LHL_glszm_ZoneEntropy	0.88848104
wavelet-LHL_glszm_ZonePercentage	0.95497672
wavelet-LHL_glszm_ZoneVariance	0.89679136
wavelet-LHL_gldm_DependenceEntropy	0.90959363
wavelet-LHL_gldm_DependenceNonUniformity	0.97523672
wavelet-LHL_gldm_DependenceNonUniformityNormalized	0.92539223
wavelet-LHL_gldm_DependenceVariance	0.93514381
wavelet-LHL_gldm_GrayLevelNonUniformity	0.97747419
wavelet-LHL_gldm_GrayLevelVariance	0.63373065
wavelet-LHL_gldm_HighGrayLevelEmphasis	0.98077222
wavelet-LHL_gldm_LargeDependenceEmphasis	0.98998297
wavelet-LHL_gldm_LargeDependenceHighGrayLevelEmphasis	0.98435478
wavelet-LHL_gldm_LargeDependenceLowGrayLevelEmphasis	0.38166416
wavelet-LHL_gldm_LowGrayLevelEmphasis	0.54432882
wavelet-LHL_gldm_SmallDependenceEmphasis	0.95739683
wavelet-LHL_gldm_SmallDependenceHighGrayLevelEmphasis	0.92406238
wavelet-LHL_gldm_SmallDependenceLowGrayLevelEmphasis	0.74232115
wavelet-LHH_firstorder_10Percentile	0.99826281
wavelet-LHH_firstorder_90Percentile	0.99826399
wavelet-LHH_firstorder_Energy	0.97416278
wavelet-LHH_firstorder_Entropy	0.98828558
wavelet-LHH_firstorder_InterquartileRange	0.99913679
wavelet-LHH_firstorder_Kurtosis	0.90858717
wavelet-LHH_firstorder_Maximum	0.9355052
wavelet-LHH_firstorder_MeanAbsoluteDeviation	0.99661839
wavelet-LHH_firstorder_Mean	0.7705724
wavelet-LHH_firstorder_Median	0.73153757
wavelet-LHH_firstorder_Minimum	0.9820041
wavelet-LHH_firstorder_Range	0.96053912
wavelet-LHH_firstorder_RobustMeanAbsoluteDeviation	0.99903199
wavelet-LHH_firstorder_RootMeanSquared	0.93269092
wavelet-LHH_firstorder_Skewness	0.51608806
wavelet-LHH_firstorder_TotalEnergy	0.97416278
wavelet-LHH_firstorder_Uniformity	0.98669997
wavelet-LHH_firstorder_Variance	0.97160351
wavelet-LHH_glcm_Autocorrelation	0.95119458
wavelet-LHH_glcm_JointAverage	0.98685381
wavelet-LHH_glcm_ClusterProminence	0.76812138
wavelet-LHH_glcm_ClusterShade	0.78144606
wavelet-LHH_glcm_ClusterTendency	0.94203642
wavelet-LHH_glcm_Contrast	0.94830413
wavelet-LHH_glcm_Correlation	0.98039679
wavelet-LHH_glcm_DifferenceAverage	0.98012234
wavelet-LHH_glcm_DifferenceEntropy	0.98883536
wavelet-LHH_glcm_DifferenceVariance	0.93785662
wavelet-LHH_glcm_JointEnergy	0.991054
wavelet-LHH_glcm_JointEntropy	0.9872971
wavelet-LHH_glcm_Imc1	0.98104042
wavelet-LHH_glcm_Imc2	0.98047963

wavelet-LHH_glcm_Idm	0.98897742
wavelet-LHH_glcm_Idmn	0.99859104
wavelet-LHH_glcm_Id	0.98949954
wavelet-LHH_glcm_Idn	0.9962595
wavelet-LHH_glcm_InverseVariance	0.98564412
wavelet-LHH_glcm_MaximumProbability	0.99511473
wavelet-LHH_glcm_SumEntropy	0.98573323
wavelet-LHH_glcm_SumSquares	0.94493709
wavelet-LHH_glrlm_GrayLevelNonUniformity	0.97514399
wavelet-LHH_glrlm_GrayLevelNonUniformityNormalized	0.98935988
wavelet-LHH_glrlm_GrayLevelVariance	0.95622284
wavelet-LHH_glrlm_HighGrayLevelRunEmphasis	0.95125327
wavelet-LHH_glrlm_LongRunEmphasis	0.98910188
wavelet-LHH_glrlm_LongRunHighGrayLevelEmphasis	0.95773778
wavelet-LHH_glrlm_LongRunLowGrayLevelEmphasis	0.99971908
wavelet-LHH_glrlm_LowGrayLevelRunEmphasis	0.99990731
wavelet-LHH_glrlm_RunEntropy	0.9690185
wavelet-LHH_glrlm_RunLengthNonUniformity	0.96994909
wavelet-LHH_glrlm_RunLengthNonUniformityNormalized	0.99210468
wavelet-LHH_glrlm_RunPercentage	0.99038966
wavelet-LHH_glrlm_RunVariance	0.98682987
wavelet-LHH_glrlm_ShortRunEmphasis	0.99160487
wavelet-LHH_glrlm_ShortRunHighGrayLevelEmphasis	0.94792219
wavelet-LHH_glrlm_ShortRunLowGrayLevelEmphasis	0.99989616
wavelet-LHH_glszm_GrayLevelNonUniformity	0.99773333
wavelet-LHH_glszm_GrayLevelNonUniformityNormalized	0.89923889
wavelet-LHH_glszm_GrayLevelVariance	0.97647574
wavelet-LHH_glszm_HighGrayLevelZoneEmphasis	0.951552
wavelet-LHH_glszm_LargeAreaEmphasis	0.83238786
wavelet-LHH_glszm_LargeAreaHighGrayLevelEmphasis	0.91791802
wavelet-LHH_glszm_LargeAreaLowGrayLevelEmphasis	0.88354476
wavelet-LHH_glszm_LowGrayLevelZoneEmphasis	0.99048651
wavelet-LHH_glszm_SizeZoneNonUniformity	0.98927738
wavelet-LHH_glszm_SizeZoneNonUniformityNormalized	0.57417135
wavelet-LHH_glszm_SmallAreaEmphasis	0.60032759
wavelet-LHH_glszm_SmallAreaHighGrayLevelEmphasis	0.9546446
wavelet-LHH_glszm_SmallAreaLowGrayLevelEmphasis	0.97588885
wavelet-LHH_glszm_ZoneEntropy	0.93764308
wavelet-LHH_glszm_ZonePercentage	0.99688816
wavelet-LHH_glszm_ZoneVariance	0.82858311
wavelet-LHH_gldm_DependenceEntropy	0.94972303
wavelet-LHH_gldm_DependenceNonUniformity	0.97627907
wavelet-LHH_gldm_DependenceNonUniformityNormalized	0.97837441
wavelet-LHH_gldm_DependenceVariance	0.96166314
wavelet-LHH_gldm_GrayLevelNonUniformity	0.97566326
wavelet-LHH_gldm_GrayLevelVariance	0.9533262
wavelet-LHH_gldm_HighGrayLevelEmphasis	0.9512246
wavelet-LHH_gldm_LargeDependenceEmphasis	0.9877206
wavelet-LHH_gldm_LargeDependenceHighGrayLevelEmphasis	0.95655206

wavelet-LHH_gldm_LargeDependenceLowGrayLevelEmphasis	0.99981884
wavelet-LHH_gldm_LowGrayLevelEmphasis	0.99991096
wavelet-LHH_gldm_SmallDependenceEmphasis	0.99697439
wavelet-LHH_gldm_SmallDependenceHighGrayLevelEmphasis	0.927819
wavelet-LHH_gldm_SmallDependenceLowGrayLevelEmphasis	0.99966591
wavelet-HLL_firstorder_10Percentile	0.95907446
wavelet-HLL_firstorder_90Percentile	0.9699012
wavelet-HLL_firstorder_Energy	0.97458827
wavelet-HLL_firstorder_Entropy	0.95066608
wavelet-HLL_firstorder_InterquartileRange	0.98855352
wavelet-HLL_firstorder_Kurtosis	0.58104328
wavelet-HLL_firstorder_Maximum	0.9822602
wavelet-HLL_firstorder_MeanAbsoluteDeviation	0.90228234
wavelet-HLL_firstorder_Mean	0.75912725
wavelet-HLL_firstorder_Median	0.96001993
wavelet-HLL_firstorder_Minimum	0.97275183
wavelet-HLL_firstorder_Range	0.9837841
wavelet-HLL_firstorder_RobustMeanAbsoluteDeviation	0.98406991
wavelet-HLL_firstorder_RootMeanSquared	0.933662
wavelet-HLL_firstorder_Skewness	0.59435092
wavelet-HLL_firstorder_TotalEnergy	0.97458827
wavelet-HLL_firstorder_Uniformity	0.96809326
wavelet-HLL_firstorder_Variance	0.69892463
wavelet-HLL_glcm_Autocorrelation	0.98413338
wavelet-HLL_glcm_JointAverage	0.97290791
wavelet-HLL_glcm_ClusterProminence	0.21883381
wavelet-HLL_glcm_ClusterShade	0.00831541
wavelet-HLL_glcm_ClusterTendency	0.6278588
wavelet-HLL_glcm_Contrast	0.60876576
wavelet-HLL_glcm_Correlation	0.95011312
wavelet-HLL_glcm_DifferenceAverage	0.88064322
wavelet-HLL_glcm_DifferenceEntropy	0.94630156
wavelet-HLL_glcm_DifferenceVariance	0.58177537
wavelet-HLL_glcm_JointEnergy	0.97752358
wavelet-HLL_glcm_JointEntropy	0.94924295
wavelet-HLL_glcm_Imc1	0.85904157
wavelet-HLL_glcm_Imc2	0.8863744
wavelet-HLL_glcm_Idm	0.97763934
wavelet-HLL_glcm_Idmn	0.96398701
wavelet-HLL_glcm_Id	0.97650899
wavelet-HLL_glcm_Idn	0.96902406
wavelet-HLL_glcm_InverseVariance	0.9078265
wavelet-HLL_glcm_MaximumProbability	0.98453696
wavelet-HLL_glcm_SumEntropy	0.9404382
wavelet-HLL_glcm_SumSquares	0.62101718
wavelet-HLL_glrlm_GrayLevelNonUniformity	0.97963833
wavelet-HLL_glrlm_GrayLevelNonUniformityNormalized	0.95833423
wavelet-HLL_glrlm_GrayLevelVariance	0.68709908
wavelet-HLL_glrlm_HighGrayLevelRunEmphasis	0.984155

wavelet-HLL_glrlm_LongRunEmphasis	0.98200819
wavelet-HLL_glrlm_LongRunHighGrayLevelEmphasis	0.97221655
wavelet-HLL_glrlm_LongRunLowGrayLevelEmphasis	0.98069872
wavelet-HLL_glrlm_LowGrayLevelRunEmphasis	0.98609382
wavelet-HLL_glrlm_RunEntropy	0.9097202
wavelet-HLL_glrlm_RunLengthNonUniformity	0.97054619
wavelet-HLL_glrlm_RunLengthNonUniformityNormalized	0.98654863
wavelet-HLL_glrlm_RunPercentage	0.99025671
wavelet-HLL_glrlm_RunVariance	0.99101
wavelet-HLL_glrlm_ShortRunEmphasis	0.98298695
wavelet-HLL_glrlm_ShortRunHighGrayLevelEmphasis	0.9847273
wavelet-HLL_glrlm_ShortRunLowGrayLevelEmphasis	0.98686417
wavelet-HLL_glszm_GrayLevelNonUniformity	0.98587161
wavelet-HLL_glszm_GrayLevelNonUniformityNormalized	0.92837692
wavelet-HLL_glszm_GrayLevelVariance	0.77184984
wavelet-HLL_glszm_HighGrayLevelZoneEmphasis	0.98466501
wavelet-HLL_glszm_LargeAreaEmphasis	0.85246896
wavelet-HLL_glszm_LargeAreaHighGrayLevelEmphasis	0.89579155
wavelet-HLL_glszm_LargeAreaLowGrayLevelEmphasis	0.90387614
wavelet-HLL_glszm_LowGrayLevelZoneEmphasis	0.98371085
wavelet-HLL_glszm_SizeZoneNonUniformity	0.9361672
wavelet-HLL_glszm_SizeZoneNonUniformityNormalized	0.96833796
wavelet-HLL_glszm_SmallAreaEmphasis	0.95840704
wavelet-HLL_glszm_SmallAreaHighGrayLevelEmphasis	0.9887273
wavelet-HLL_glszm_SmallAreaLowGrayLevelEmphasis	0.96604001
wavelet-HLL_glszm_ZoneEntropy	0.9073489
wavelet-HLL_glszm_ZonePercentage	0.96351172
wavelet-HLL_glszm_ZoneVariance	0.85242996
wavelet-HLL_gldm_DependenceEntropy	0.92433532
wavelet-HLL_gldm_DependenceNonUniformity	0.97868279
wavelet-HLL_gldm_DependenceNonUniformityNormalized	0.93661302
wavelet-HLL_gldm_DependenceVariance	0.93314596
wavelet-HLL_gldm_GrayLevelNonUniformity	0.98090215
wavelet-HLL_gldm_GrayLevelVariance	0.69846569
wavelet-HLL_gldm_HighGrayLevelEmphasis	0.98408676
wavelet-HLL_gldm_LargeDependenceEmphasis	0.99330482
wavelet-HLL_gldm_LargeDependenceHighGrayLevelEmphasis	0.98030748
wavelet-HLL_gldm_LargeDependenceLowGrayLevelEmphasis	0.98412324
wavelet-HLL_gldm_LowGrayLevelEmphasis	0.98612465
wavelet-HLL_gldm_SmallDependenceEmphasis	0.965653
wavelet-HLL_gldm_SmallDependenceHighGrayLevelEmphasis	0.95192743
wavelet-HLL_gldm_SmallDependenceLowGrayLevelEmphasis	0.98631154
wavelet-HLH_firstorder_10Percentile	0.99877697
wavelet-HLH_firstorder_90Percentile	0.99891463
wavelet-HLH_firstorder_Energy	0.97415841
wavelet-HLH_firstorder_Entropy	0.99188277
wavelet-HLH_firstorder_InterquartileRange	0.99938823
wavelet-HLH_firstorder_Kurtosis	0.90478192
wavelet-HLH_firstorder_Maximum	0.99507853

wavelet-HLH_firstorder_MeanAbsoluteDeviation	0.99739156
wavelet-HLH_firstorder_Mean	0.73426424
wavelet-HLH_firstorder_Median	0.58569686
wavelet-HLH_firstorder_Minimum	0.99768642
wavelet-HLH_firstorder_Range	0.9969244
wavelet-HLH_firstorder_RobustMeanAbsoluteDeviation	0.99928642
wavelet-HLH_firstorder_RootMeanSquared	0.95010328
wavelet-HLH_firstorder_Skewness	-0.1864321
wavelet-HLH_firstorder_TotalEnergy	0.97415841
wavelet-HLH_firstorder_Uniformity	0.99107806
wavelet-HLH_firstorder_Variance	0.98377872
wavelet-HLH_glcm_Autocorrelation	0.99974151
wavelet-HLH_glcm_JointAverage	0.9984895
wavelet-HLH_glcm_ClusterProminence	0.95617358
wavelet-HLH_glcm_ClusterShade	0.87986626
wavelet-HLH_glcm_ClusterTendency	0.96296732
wavelet-HLH_glcm_Contrast	0.96625517
wavelet-HLH_glcm_Correlation	0.96163051
wavelet-HLH_glcm_DifferenceAverage	0.98417697
wavelet-HLH_glcm_DifferenceEntropy	0.99104409
wavelet-HLH_glcm_DifferenceVariance	0.96239473
wavelet-HLH_glcm_JointEnergy	0.99290512
wavelet-HLH_glcm_JointEntropy	0.99015864
wavelet-HLH_glcm_Imc1	0.97687439
wavelet-HLH_glcm_Imc2	0.98574167
wavelet-HLH_glcm_Idm	0.98998356
wavelet-HLH_glcm_Idmn	0.90427153
wavelet-HLH_glcm_Id	0.9895214
wavelet-HLH_glcm_Idn	0.94779845
wavelet-HLH_glcm_InverseVariance	0.97096305
wavelet-HLH_glcm_MaximumProbability	0.99219696
wavelet-HLH_glcm_SumEntropy	0.98829603
wavelet-HLH_glcm_SumSquares	0.96456192
wavelet-HLH_glrlm_GrayLevelNonUniformity	0.97601449
wavelet-HLH_glrlm_GrayLevelNonUniformityNormalized	0.99285441
wavelet-HLH_glrlm_GrayLevelVariance	0.97888834
wavelet-HLH_glrlm_HighGrayLevelRunEmphasis	0.99974415
wavelet-HLH_glrlm_LongRunEmphasis	0.98560959
wavelet-HLH_glrlm_LongRunHighGrayLevelEmphasis	0.99913955
wavelet-HLH_glrlm_LongRunLowGrayLevelEmphasis	0.93951638
wavelet-HLH_glrlm_LowGrayLevelRunEmphasis	0.94113408
wavelet-HLH_glrlm_RunEntropy	0.97188713
wavelet-HLH_glrlm_RunLengthNonUniformity	0.97198034
wavelet-HLH_glrlm_RunLengthNonUniformityNormalized	0.98989068
wavelet-HLH_glrlm_RunPercentage	0.98753829
wavelet-HLH_glrlm_RunVariance	0.98097552
wavelet-HLH_glrlm_ShortRunEmphasis	0.98947661
wavelet-HLH_glrlm_ShortRunHighGrayLevelEmphasis	0.99980367
wavelet-HLH_glrlm_ShortRunLowGrayLevelEmphasis	0.94142924

wavelet-HLH_glszm_GrayLevelNonUniformity	0.9978738
wavelet-HLH_glszm_GrayLevelNonUniformityNormalized	0.93415504
wavelet-HLH_glszm_GrayLevelVariance	0.99156559
wavelet-HLH_glszm_HighGrayLevelZoneEmphasis	0.99956765
wavelet-HLH_glszm_LargeAreaEmphasis	0.87258084
wavelet-HLH_glszm_LargeAreaHighGrayLevelEmphasis	0.84828763
wavelet-HLH_glszm_LargeAreaLowGrayLevelEmphasis	0.87651992
wavelet-HLH_glszm_LowGrayLevelZoneEmphasis	0.93192181
wavelet-HLH_glszm_SizeZoneNonUniformity	0.99048852
wavelet-HLH_glszm_SizeZoneNonUniformityNormalized	0.57923177
wavelet-HLH_glszm_SmallAreaEmphasis	0.53951245
wavelet-HLH_glszm_SmallAreaHighGrayLevelEmphasis	0.99966489
wavelet-HLH_glszm_SmallAreaLowGrayLevelEmphasis	0.94713549
wavelet-HLH_glszm_ZoneEntropy	0.96290704
wavelet-HLH_glszm_ZonePercentage	0.99749955
wavelet-HLH_glszm_ZoneVariance	0.86377255
wavelet-HLH_gldm_DependenceEntropy	0.95186428
wavelet-HLH_gldm_DependenceNonUniformity	0.97859915
wavelet-HLH_gldm_DependenceNonUniformityNormalized	0.97584346
wavelet-HLH_gldm_DependenceVariance	0.95794796
wavelet-HLH_gldm_GrayLevelNonUniformity	0.97566564
wavelet-HLH_gldm_GrayLevelVariance	0.97639918
wavelet-HLH_gldm_HighGrayLevelEmphasis	0.99974236
wavelet-HLH_gldm_LargeDependenceEmphasis	0.9828733
wavelet-HLH_gldm_LargeDependenceHighGrayLevelEmphasis	0.99938939
wavelet-HLH_gldm_LargeDependenceLowGrayLevelEmphasis	0.93926388
wavelet-HLH_gldm_LowGrayLevelEmphasis	0.9408161
wavelet-HLH_gldm_SmallDependenceEmphasis	0.99778026
wavelet-HLH_gldm_SmallDependenceHighGrayLevelEmphasis	0.99847998
wavelet-HLH_gldm_SmallDependenceLowGrayLevelEmphasis	0.93938684
wavelet-HHL_firstorder_10Percentile	0.98305739
wavelet-HHL_firstorder_90Percentile	0.98040444
wavelet-HHL_firstorder_Energy	0.97415187
wavelet-HHL_firstorder_Entropy	0.94728163
wavelet-HHL_firstorder_InterquartileRange	0.9936745
wavelet-HHL_firstorder_Kurtosis	0.98213735
wavelet-HHL_firstorder_Maximum	0.92880872
wavelet-HHL_firstorder_MeanAbsoluteDeviation	0.92215078
wavelet-HHL_firstorder_Mean	0.58908992
wavelet-HHL_firstorder_Median	0.93482391
wavelet-HHL_firstorder_Minimum	0.96108298
wavelet-HHL_firstorder_Range	0.95645507
wavelet-HHL_firstorder_RobustMeanAbsoluteDeviation	0.9918747
wavelet-HHL_firstorder_RootMeanSquared	0.05973877
wavelet-HHL_firstorder_Skewness	0.63741815
wavelet-HHL_firstorder_TotalEnergy	0.97415187
wavelet-HHL_firstorder_Uniformity	0.96013868
wavelet-HHL_firstorder_Variance	0.74322311
wavelet-HHL_glcm_Autocorrelation	0.97467886

wavelet-HHL_glcm_JointAverage	0.95855504
wavelet-HHL_glcm_ClusterProminence	0.41274503
wavelet-HHL_glcm_ClusterShade	-0.67685697
wavelet-HHL_glcm_ClusterTendency	0.66369221
wavelet-HHL_glcm_Contrast	0.63674865
wavelet-HHL_glcm_Correlation	0.97574381
wavelet-HHL_glcm_DifferenceAverage	0.84517269
wavelet-HHL_glcm_DifferenceEntropy	0.93628822
wavelet-HHL_glcm_DifferenceVariance	0.61811841
wavelet-HHL_glcm_JointEnergy	0.97071733
wavelet-HHL_glcm_JointEntropy	0.93987072
wavelet-HHL_glcm_Imc1	0.88463961
wavelet-HHL_glcm_Imc2	0.9085728
wavelet-HHL_glcm_Idm	0.95540845
wavelet-HHL_glcm_Idmn	0.81192157
wavelet-HHL_glcm_Id	0.95468596
wavelet-HHL_glcm_Idn	0.89870902
wavelet-HHL_glcm_InverseVariance	0.87627623
wavelet-HHL_glcm_MaximumProbability	0.97267196
wavelet-HHL_glcm_SumEntropy	0.93433522
wavelet-HHL_glcm_SumSquares	0.65125886
wavelet-HHL_glrlm_GrayLevelNonUniformity	0.97712055
wavelet-HHL_glrlm_GrayLevelNonUniformityNormalized	0.96422689
wavelet-HHL_glrlm_GrayLevelVariance	0.75000599
wavelet-HHL_glrlm_HighGrayLevelRunEmphasis	0.97409559
wavelet-HHL_glrlm_LongRunEmphasis	0.98807537
wavelet-HHL_glrlm_LongRunHighGrayLevelEmphasis	0.9795419
wavelet-HHL_glrlm_LongRunLowGrayLevelEmphasis	0.74393436
wavelet-HHL_glrlm_LowGrayLevelRunEmphasis	0.76496114
wavelet-HHL_glrlm_RunEntropy	0.92090099
wavelet-HHL_glrlm_RunLengthNonUniformity	0.96758357
wavelet-HHL_glrlm_RunLengthNonUniformityNormalized	0.97829452
wavelet-HHL_glrlm_RunPercentage	0.98514025
wavelet-HHL_glrlm_RunVariance	0.99146699
wavelet-HHL_glrlm_ShortRunEmphasis	0.98307702
wavelet-HHL_glrlm_ShortRunHighGrayLevelEmphasis	0.96922585
wavelet-HHL_glrlm_ShortRunLowGrayLevelEmphasis	0.77227487
wavelet-HHL_glszm_GrayLevelNonUniformity	0.98801539
wavelet-HHL_glszm_GrayLevelNonUniformityNormalized	0.98183786
wavelet-HHL_glszm_GrayLevelVariance	0.86026597
wavelet-HHL_glszm_HighGrayLevelZoneEmphasis	0.96608723
wavelet-HHL_glszm_LargeAreaEmphasis	0.97688666
wavelet-HHL_glszm_LargeAreaHighGrayLevelEmphasis	0.93391991
wavelet-HHL_glszm_LargeAreaLowGrayLevelEmphasis	0.65755599
wavelet-HHL_glszm_LowGrayLevelZoneEmphasis	0.72638757
wavelet-HHL_glszm_SizeZoneNonUniformity	0.96598059
wavelet-HHL_glszm_SizeZoneNonUniformityNormalized	0.99540458
wavelet-HHL_glszm_SmallAreaEmphasis	0.99606888
wavelet-HHL_glszm_SmallAreaHighGrayLevelEmphasis	0.96387931

wavelet-HHL_glszm_SmallAreaLowGrayLevelEmphasis	0.52243181
wavelet-HHL_glszm_ZoneEntropy	0.95169548
wavelet-HHL_glszm_ZonePercentage	0.97261358
wavelet-HHL_glszm_ZoneVariance	0.97688773
wavelet-HHL_gldm_DependenceEntropy	0.93746757
wavelet-HHL_gldm_DependenceNonUniformity	0.97718546
wavelet-HHL_gldm_DependenceNonUniformityNormalized	0.95761919
wavelet-HHL_gldm_DependenceVariance	0.96449448
wavelet-HHL_gldm_GrayLevelNonUniformity	0.97763789
wavelet-HHL_gldm_GrayLevelVariance	0.73917306
wavelet-HHL_gldm_HighGrayLevelEmphasis	0.97442649
wavelet-HHL_gldm_LargeDependenceEmphasis	0.98910867
wavelet-HHL_gldm_LargeDependenceHighGrayLevelEmphasis	0.97951151
wavelet-HHL_gldm_LargeDependenceLowGrayLevelEmphasis	0.75142176
wavelet-HHL_gldm_LowGrayLevelEmphasis	0.76364184
wavelet-HHL_gldm_SmallDependenceEmphasis	0.97329157
wavelet-HHL_gldm_SmallDependenceHighGrayLevelEmphasis	0.90253631
wavelet-HHL_gldm_SmallDependenceLowGrayLevelEmphasis	0.82679385
wavelet-HHH_firstorder_10Percentile	0.99907011
wavelet-HHH_firstorder_90Percentile	0.99911974
wavelet-HHH_firstorder_Energy	0.97417223
wavelet-HHH_firstorder_Entropy	0.98272578
wavelet-HHH_firstorder_InterquartileRange	0.99946439
wavelet-HHH_firstorder_Kurtosis	0.93278297
wavelet-HHH_firstorder_Maximum	0.99960813
wavelet-HHH_firstorder_MeanAbsoluteDeviation	0.99846221
wavelet-HHH_firstorder_Mean	0.84210252
wavelet-HHH_firstorder_Median	-0.07145588
wavelet-HHH_firstorder_Minimum	0.99893019
wavelet-HHH_firstorder_Range	0.99941538
wavelet-HHH_firstorder_RobustMeanAbsoluteDeviation	0.99938867
wavelet-HHH_firstorder_RootMeanSquared	0.98348471
wavelet-HHH_firstorder_Skewness	0.08415438
wavelet-HHH_firstorder_TotalEnergy	0.97417223
wavelet-HHH_firstorder_Uniformity	0.97673516
wavelet-HHH_firstorder_Variance	0.98776266
wavelet-HHH_glcm_Autocorrelation	1
wavelet-HHH_glcm_JointAverage	0.99999999
wavelet-HHH_glcm_ClusterProminence	0.87506412
wavelet-HHH_glcm_ClusterShade	0.92294052
wavelet-HHH_glcm_ClusterTendency	0.9440938
wavelet-HHH_glcm_Contrast	0.94786632
wavelet-HHH_glcm_Correlation	0.97960662
wavelet-HHH_glcm_DifferenceAverage	0.96829434
wavelet-HHH_glcm_DifferenceEntropy	0.98182866
wavelet-HHH_glcm_DifferenceVariance	0.94148432
wavelet-HHH_glcm_JointEnergy	0.98386063
wavelet-HHH_glcm_JointEntropy	0.98012884
wavelet-HHH_glcm_Imc1	0.99189559

wavelet-HHH_glcm_Imc2	0.99120976
wavelet-HHH_glcm_Idm	0.97701778
wavelet-HHH_glcm_Idmn	0.99999504
wavelet-HHH_glcm_Id	0.97814954
wavelet-HHH_glcm_Idn	0.99998571
wavelet-HHH_glcm_InverseVariance	0.97930308
wavelet-HHH_glcm_MaximumProbability	0.99697568
wavelet-HHH_glcm_SumEntropy	0.98020657
wavelet-HHH_glcm_SumSquares	0.94585143
wavelet-HHH_glrlm_GrayLevelNonUniformity	0.9747844
wavelet-HHH_glrlm_GrayLevelNonUniformityNormalized	0.9789992
wavelet-HHH_glrlm_GrayLevelVariance	0.96063058
wavelet-HHH_glrlm_HighGrayLevelRunEmphasis	0.99999988
wavelet-HHH_glrlm_LongRunEmphasis	0.97783615
wavelet-HHH_glrlm_LongRunHighGrayLevelEmphasis	0.99998752
wavelet-HHH_glrlm_LongRunLowGrayLevelEmphasis	0.99977042
wavelet-HHH_glrlm_LowGrayLevelRunEmphasis	0.99999908
wavelet-HHH_glrlm_RunEntropy	0.94995627
wavelet-HHH_glrlm_RunLengthNonUniformity	0.97290442
wavelet-HHH_glrlm_RunLengthNonUniformityNormalized	0.9818378
wavelet-HHH_glrlm_RunPercentage	0.97181579
wavelet-HHH_glrlm_RunVariance	0.96989473
wavelet-HHH_glrlm_ShortRunEmphasis	0.97683013
wavelet-HHH_glrlm_ShortRunHighGrayLevelEmphasis	0.9999983
wavelet-HHH_glrlm_ShortRunLowGrayLevelEmphasis	0.99997299
wavelet-HHH_glszm_GrayLevelNonUniformity	0.98908165
wavelet-HHH_glszm_GrayLevelNonUniformityNormalized	0.75780305
wavelet-HHH_glszm_GrayLevelVariance	0.98292221
wavelet-HHH_glszm_HighGrayLevelZoneEmphasis	0.99970275
wavelet-HHH_glszm_LargeAreaEmphasis	0.5767565
wavelet-HHH_glszm_LargeAreaHighGrayLevelEmphasis	0.47411598
wavelet-HHH_glszm_LargeAreaLowGrayLevelEmphasis	0.62209605
wavelet-HHH_glszm_LowGrayLevelZoneEmphasis	0.86370196
wavelet-HHH_glszm_SizeZoneNonUniformity	0.98822527
wavelet-HHH_glszm_SizeZoneNonUniformityNormalized	-0.081538
wavelet-HHH_glszm_SmallAreaEmphasis	0.18516456
wavelet-HHH_glszm_SmallAreaHighGrayLevelEmphasis	0.99834654
wavelet-HHH_glszm_SmallAreaLowGrayLevelEmphasis	0.4945677
wavelet-HHH_glszm_ZoneEntropy	0.62400121
wavelet-HHH_glszm_ZonePercentage	0.99462698
wavelet-HHH_glszm_ZoneVariance	0.5797234
wavelet-HHH_gldm_DependenceEntropy	0.96807836
wavelet-HHH_gldm_DependenceNonUniformity	0.97314293
wavelet-HHH_gldm_DependenceNonUniformityNormalized	0.95575824
wavelet-HHH_gldm_DependenceVariance	0.98796201
wavelet-HHH_gldm_GrayLevelNonUniformity	0.97474116
wavelet-HHH_gldm_GrayLevelVariance	0.95900302
wavelet-HHH_gldm_HighGrayLevelEmphasis	0.99999998
wavelet-HHH_gldm_LargeDependenceEmphasis	0.96768591

wavelet-HHH_gldm_LargeDependenceHighGrayLevelEmphasis	0.99999483
wavelet-HHH_gldm_LargeDependenceLowGrayLevelEmphasis	0.99986781
wavelet-HHH_gldm_LowGrayLevelEmphasis	0.99999968
wavelet-HHH_gldm_SmallDependenceEmphasis	0.99707604
wavelet-HHH_gldm_SmallDependenceHighGrayLevelEmphasis	0.99889727
wavelet-HHH_gldm_SmallDependenceLowGrayLevelEmphasis	0.99951357
wavelet-LLL_firstorder_10Percentile	0.66642136
wavelet-LLL_firstorder_90Percentile	0.8014558
wavelet-LLL_firstorder_Energy	0.97477261
wavelet-LLL_firstorder_Entropy	0.85605819
wavelet-LLL_firstorder_InterquartileRange	0.90762628
wavelet-LLL_firstorder_Kurtosis	0.51432835
wavelet-LLL_firstorder_Maximum	0.85028575
wavelet-LLL_firstorder_MeanAbsoluteDeviation	0.61880729
wavelet-LLL_firstorder_Mean	0.76521692
wavelet-LLL_firstorder_Median	0.9933299
wavelet-LLL_firstorder_Minimum	0.74160147
wavelet-LLL_firstorder_Range	0.87461906
wavelet-LLL_firstorder_RobustMeanAbsoluteDeviation	0.84275402
wavelet-LLL_firstorder_RootMeanSquared	0.67995659
wavelet-LLL_firstorder_Skewness	0.75170357
wavelet-LLL_firstorder_TotalEnergy	0.97477261
wavelet-LLL_firstorder_Uniformity	0.90408815
wavelet-LLL_firstorder_Variance	0.53607868
wavelet-LLL_glcm_Autocorrelation	0.82029051
wavelet-LLL_glcm_JointAverage	0.74271431
wavelet-LLL_glcm_ClusterProminence	0.31312667
wavelet-LLL_glcm_ClusterShade	0.23209494
wavelet-LLL_glcm_ClusterTendency	0.44666525
wavelet-LLL_glcm_Contrast	0.59905345
wavelet-LLL_glcm_Correlation	0.55526543
wavelet-LLL_glcm_DifferenceAverage	0.79965563
wavelet-LLL_glcm_DifferenceEntropy	0.88384337
wavelet-LLL_glcm_DifferenceVariance	0.59170584
wavelet-LLL_glcm_JointEnergy	0.94687104
wavelet-LLL_glcm_JointEntropy	0.88352343
wavelet-LLL_glcm_Imc1	0.90845641
wavelet-LLL_glcm_Imc2	0.79585646
wavelet-LLL_glcm_Idm	0.93890383
wavelet-LLL_glcm_Idmn	0.97952159
wavelet-LLL_glcm_Id	0.93818839
wavelet-LLL_glcm_Idn	0.96245084
wavelet-LLL_glcm_InverseVariance	0.90719162
wavelet-LLL_glcm_MaximumProbability	0.97691893
wavelet-LLL_glcm_SumEntropy	0.84721505
wavelet-LLL_glcm_SumSquares	0.46770431
wavelet-LLL_glrlm_GrayLevelNonUniformity	0.97505348
wavelet-LLL_glrlm_GrayLevelNonUniformityNormalized	0.84218189
wavelet-LLL_glrlm_GrayLevelVariance	0.51436333

wavelet-LLL_glrlm_HighGrayLevelRunEmphasis	0.82175721
wavelet-LLL_glrlm_LongRunEmphasis	0.96048708
wavelet-LLL_glrlm_LongRunHighGrayLevelEmphasis	0.53010803
wavelet-LLL_glrlm_LongRunLowGrayLevelEmphasis	0.90843526
wavelet-LLL_glrlm_LowGrayLevelRunEmphasis	0.95522234
wavelet-LLL_glrlm_RunEntropy	0.79782909
wavelet-LLL_glrlm_RunLengthNonUniformity	0.95953863
wavelet-LLL_glrlm_RunLengthNonUniformityNormalized	0.94452174
wavelet-LLL_glrlm_RunPercentage	0.96666662
wavelet-LLL_glrlm_RunVariance	0.97762549
wavelet-LLL_glrlm_ShortRunEmphasis	0.93504583
wavelet-LLL_glrlm_ShortRunHighGrayLevelEmphasis	0.84755957
wavelet-LLL_glrlm_ShortRunLowGrayLevelEmphasis	0.96187907
wavelet-LLL_glszm_GrayLevelNonUniformity	0.95402235
wavelet-LLL_glszm_GrayLevelNonUniformityNormalized	0.85194869
wavelet-LLL_glszm_GrayLevelVariance	0.72034686
wavelet-LLL_glszm_HighGrayLevelZoneEmphasis	0.83575043
wavelet-LLL_glszm_LargeAreaEmphasis	0.76170296
wavelet-LLL_glszm_LargeAreaHighGrayLevelEmphasis	0.76206027
wavelet-LLL_glszm_LargeAreaLowGrayLevelEmphasis	0.42650988
wavelet-LLL_glszm_LowGrayLevelZoneEmphasis	0.97142981
wavelet-LLL_glszm_SizeZoneNonUniformity	0.86375099
wavelet-LLL_glszm_SizeZoneNonUniformityNormalized	0.9206974
wavelet-LLL_glszm_SmallAreaEmphasis	0.93112397
wavelet-LLL_glszm_SmallAreaHighGrayLevelEmphasis	0.86036296
wavelet-LLL_glszm_SmallAreaLowGrayLevelEmphasis	0.96398976
wavelet-LLL_glszm_ZoneEntropy	0.77877134
wavelet-LLL_glszm_ZonePercentage	0.88639678
wavelet-LLL_glszm_ZoneVariance	0.76162756
wavelet-LLL_gldm_DependenceEntropy	0.85263881
wavelet-LLL_gldm_DependenceNonUniformity	0.97916207
wavelet-LLL_gldm_DependenceNonUniformityNormalized	0.9774416
wavelet-LLL_gldm_DependenceVariance	0.95936917
wavelet-LLL_gldm_GrayLevelNonUniformity	0.98098356
wavelet-LLL_gldm_GrayLevelVariance	0.53602104
wavelet-LLL_gldm_HighGrayLevelEmphasis	0.82066268
wavelet-LLL_gldm_LargeDependenceEmphasis	0.97857249
wavelet-LLL_gldm_LargeDependenceHighGrayLevelEmphasis	0.58937859
wavelet-LLL_gldm_LargeDependenceLowGrayLevelEmphasis	0.91111801
wavelet-LLL_gldm_LowGrayLevelEmphasis	0.95348114
wavelet-LLL_gldm_SmallDependenceEmphasis	0.89316526
wavelet-LLL_gldm_SmallDependenceHighGrayLevelEmphasis	0.84526569
wavelet-LLL_gldm_SmallDependenceLowGrayLevelEmphasis	0.97112508
Mean	0.889723667
