# Supplementary material for: Diagnosis of Acute Aortic Syndromes on Non-Contrast CT Images with Radiomics-Based Machine Learning
Source: Biology (Basel). 2023 Feb 21;12(3):337. doi: 10.3390/biology12030337 (PMC10045362; doi:10.3390/biology12030337)

Table 4. Performance of the detection models in the validation cohort.

Models	Validation cohort (N=49)			
	ACC (95% CI)	Sensitivity (95% CI)	Specificity (95% CI)	AUC (95% CI)
XGB	0.919(0.846,1.00)	0.857(0.616,1.00)	0.963(0.884,1.00)	0.985(0.926,1.00)
RF	0.946(0.866,1.00)	0.846(0.606,1.00)	1.00(0.921,1.00)	0.991(0.963,1.00)
LG	0.973(0.897,1.00)	0.917(0.736,1.00)	1.00(0.923,1.00)	0.991(0.961,1.00)
GBDT	0.919(0.834,1.00)	0.833(0.594,1.00)	0.963(0.873,1.00)	0.984(0.912,1.00)
SVM	0.946(0.877,1.00)	0.900(0.696,1.00)	0.964(0.903,1.00)	0.993(0.965,1.00)
DT	0.919(0.823,0.989)	0.818(0.575,1.00)	0.958(0.853,1.00)	0.883(0.760,0.999)
KNN	0.919(0.835,0.997)	0.769(0.497,1.00)	1.00(0.927,1.00)	0.875(0.734,0.995)
GNB	0.946(0.872,1.00)	0.829(0.604,1.00)	1.00(0.938,1.00)	0.987(0.952,1.00)

ACC, accuracy; CI, confidence interval; XGB, eXtreme Gradient Boostin; RF, Random Forest; LG, logistic regression; GBDT, Gradient Boosting Decision Tree; SVM, Support Vector Machine; DT, decision tree; KNN, K-nearest-neighbor; GNB, GaussianNB

Table 5. Performance of the detection models in the internal and external testing cohorts.

Models	Internal testing cohort (n=46)				External testing cohort (n=95)
	ACC (95% CI)	Sensitivity (95% CI)	Specificity (95% CI)	AUC (95% CI)	ACC (95% CI)
XGB	0.935(0.890,0.989)	0.778(0.583,0.912)	1.00(0.944,1.00)	0.982(0.952,1.00)	0.990(0.883,1.00)
RF	0.935(0.895,0.984)	0.778(0.612,0.908)	1.00(0.939,1.00)	0.982(0.956,1.00)	0.990(0.858,1.00)
LG	0.978(0.967,0.987)	0.889(0.861,0.913)	1.00(0.988,1.00)	0.991(0.976,1.00)	0.979(0.824,1.00)
GBDT	0.891(0.836,0.943)	0.667(0.560,0.813)	0.946(0.881,0.996)	0.928(0.861,0.981)	0.979(0.796,1.00)
SVM	0.957(0.945,0.988)	0.889(0.888,0.889)	0.973(0.959,1.00)	0.997(0.992,1.00)	0.991(0.937,1.00)
DT	0.870(0.806,0.930)	0.667(0.563,0.769)	0.919(0.846,0.992)	0.792(0.727,0.853)	0.811(0.543,1.00)
KNN	0.935(0.935,0.970)	0.667(0.584,0.846)	1.00(0.990,1.00)	0.986(0.934,1.00)	0.505(0.541,0.640)
GNB	0.946(0.971,0.984)	0.889(0.879,0.899)	1.00(0.992,1.00)	0.982(0.962,0.996)	0.905(0.875,0.922)

ACC, accuracy; CI, confidence interval; XGB, eXtreme Gradient Boostin; RF, Random Forest; LG, logistic regression; GBDT, Gradient Boosting Decision Tree; SVM, Support Vector Machine; DT, decision tree; KNN, K-nearest-neighbor; GNB, GaussianNB

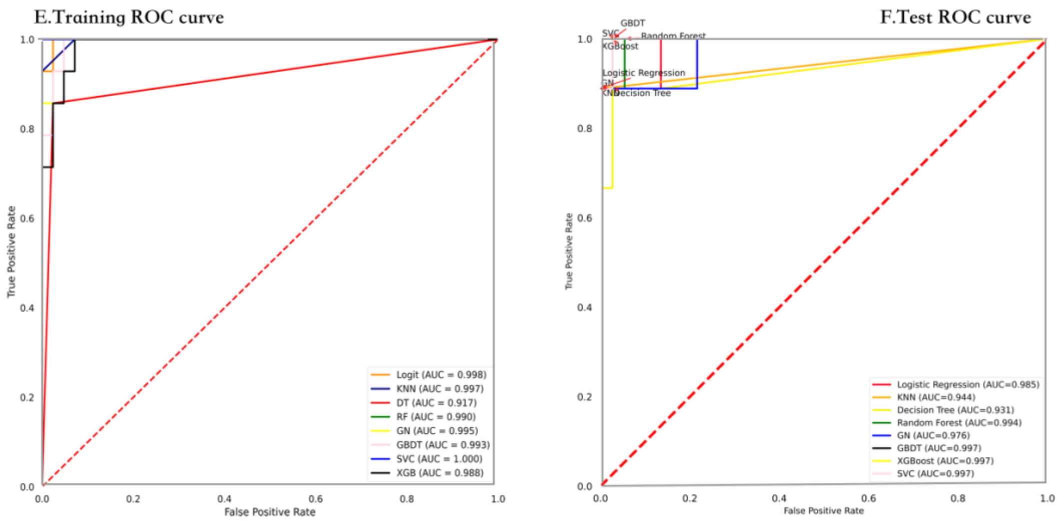

Supplement: Supplementary file 1 [file biology-12-00337-s001.zip › SupplementaryS2.pdf]
